# Antimicrobial Activity of Non-steroidal Anti-inflammatory Drugs on Biofilm: Current Evidence and Potential for Drug Repurposing

**DOI:** 10.3389/fmicb.2021.707629

**Published:** 2021-07-27

**Authors:** Rodrigo Cuiabano Paes Leme, Raquel Bandeira da Silva

**Affiliations:** ^1^Laboratório Especial de Microbiologia Clínica (LEMC), Universidade Federal de São Paulo, Escola Paulista de Medicina, São Paulo, Brazil; ^2^Department of Infectious Diseases, Centro Universitário de Volta Redonda, Volta Redonda, Brazil; ^3^Department of Internal Medicine, Hospital São Francisco de Assis, Belo Horizonte, Brazil

**Keywords:** non-steroidal anti-inflammatory drugs, biofilm, *Staphylococcus aureus*, gram-negative pathogens, antibiotic resistance

## Abstract

It has been demonstrated that some non-steroidal anti-inflammatory drugs (NSAIDs), like acetylsalicylic acid, diclofenac, and ibuprofen, have anti-biofilm activity in concentrations found in human pharmacokinetic studies, which could fuel an interest in repurposing these well tolerated drugs as adjunctive therapies for biofilm-related infections. Here we sought to review the currently available data on the anti-biofilm activity of NSAIDs and its relevance in a clinical context. We performed a systematic literature review to identify the most commonly tested NSAIDs drugs in the last 5 years, the bacterial species that have demonstrated to be responsive to their actions, and the emergence of resistance to these molecules. We found that most studies investigating NSAIDs’ activity against biofilms were *in vitro*, and frequently tested non-clinical bacterial isolates, which may not adequately represent the bacterial populations that cause clinically-relevant biofilm-related infections. Furthermore, studies concerning NSAIDs and antibiotic resistance are scarce, with divergent outcomes. Although the potential to use NSAIDs to control biofilm-related infections seems to be an exciting avenue, there is a paucity of studies that tested these drugs using appropriate *in vivo* models of biofilm infections or in controlled human clinical trials to support their repurposing as anti-biofilm agents.

## Introduction

Biofilm formation is a common mechanism that promotes the survival and persistence of medically important bacteria in different abiotic and biotic niches. Approximately 80% of chronic and recurrent bacterial infections in humans are related to biofilms, some of which associated with high mortality and morbidity (Banerjee et al., 2020; Uruén et al., 2020). Biofilms are defined as a community of sessile microbes held together by a secreted matrix of polysaccharides, proteins, and extracellular DNA, adherent to a surface, interface, or other cells. It serves as a natural physical barrier, and harbor other remarkable functions: they establish a constant flow of nutrients for microbial growth, create conditions for interbacterial interaction, promote the exchange of genetic material, resistance to extreme pH, and can become significantly more resistant to the action of antimicrobial agents and immune defenses, making biofilm-related infections challenging to eradicate (Yin et al., 2019; Salgar-Chaparro et al., 2020; Uruén et al., 2020). As a result, most healthcare-related infections associated with the insertion of an artificial medical device can only be controlled after removing the device, which results in more challenging and expensive care of these patients (Uruén et al., 2020).

An increasing body of evidence suggests that non-steroidal anti-inflammatory drugs (NSAIDs) seem to exert some antimicrobial and anti-biofilm activities against clinically relevant bacteria. Since these drugs are already universally used in medicine, novel indications to overcome currently unmet problems, like augmenting eradication of biofilm-related infections, seem to be attractive. Here, we present a short discussion on the potential of NSAID to control biofilm infections, focusing on *Staphylococcus aureus* and selected Gram-negative pathogens, as well as currently identified drawbacks that in our opinion hinder the potential to repurposing NSAIDs as anti-biofilm agents. We performed a systematic literature search to identify the most commonly tested NSAIDs drugs in the last 5 years, the bacterial species that have demonstrated to be responsive to their actions, and the emergence of resistance to these molecules.

However, although the available *in vitro* data can create some enthusiasm on the prospects of repurposing NSAIDs as anti-biofilm agents, these observations can hardly be translated into clinical practice as it stands. Data on NSAIDs’ *in vitro* activity against planktonic bacteria and biofilms are not consistent (Riordan et al., 2011; Belfield et al., 2017; Abbas et al., 2020; Gerner et al., 2020; Leão et al., 2020). In addition, there is evidence showing the selection of resistant bacterial populations following exposure to NSAIDs (Cohen et al., 1993; Gustafson et al., 1999; Helal, 2012; Belfield et al., 2017; Verma et al., 2018).

## Methods

A systematic search in PubMed and Scopus (Supplementary Appendix A), was conducted by one the authors. Subsequently, all authors analyzed the abstracts and extracted data from the manuscripts. This search aimed to quantify the number of studies that tested NSAIDs’ activities against biofilm-forming pathogens *in vitro* or *in vivo and* identify the most targeted organisms in reports published in the last 5 years. Thirty two studies were found. They were all experimental *in vitro* studies, except for one, which investigate whether aspirin (ASA, acetylsalicylic acid) could promote infection control for patients with periprosthetic joint infections (Wei et al., 2020). Most of them tested pathogens that are highly relevant in biofilm-associated infections (Table 1). Moreover, we performed a bibliometric analysis to determine the most studied NSAIDs regarding biofilm activity also in the last 5 years (Table 2). *Bibliometric analysis* is a universally used instrument that applies mathematical and statistical methods to assess research trends and progress. For citations, the data were analyzed from a Microsoft Excel file containing all Digital Object Identifiers (DOI) using VOS viewer software (version 1.6.15) through exported PubMed citation files. In addition, we manually added other studies from a Scopus systematic search.

**TABLE 1:** Studied human pathogens in the biofilm state concerning non-steroidal anti-inflammatory drugs (NSAIDs) activity in the last 5 years.

Biofilm-forming pathogen	Number of experimental studies
*Pseudomonas aeruginosa*	Soheili et al., 2015; Belfield et al., 2017; Fourie et al., 2017; Ulusoy et al., 2017; Pereira et al., 2018; She et al., 2018; Abidi et al., 2019; Dai et al., 2019; Gerner et al., 2020
*Staphylococcus aureus*	Belfield et al., 2017; Dotto et al., 2017; Xu et al., 2017; Cai et al., 2018; Oliveira et al., 2019; Abbas et al., 2020; Tzeng et al., 2020
*Candida* spp.	Rosato et al., 2016; Fourie et al., 2017; Brilhante et al., 2020; Lin et al., 2020; Santos et al., 2020
*Escherichia coli*	Cattò et al., 2017; Pereira et al., 2018; Leão et al., 2020
*Staphylococcus epidermidis*	Pereira et al., 2018; Abidi et al., 2019
*Enterococcus faecalis*	Pereira et al., 2018
*Enterococcus faecium*	Pereira et al., 2018
*Acinetobacter baumannii*	Pereira et al., 2018
*Klebsiella pneumoniae*	Pereira et al., 2018
*Enterobacter* spp.	Pereira et al., 2018
*Staphylococcus xylosus*	Xu et al., 2017
*Trichosporon asahii*	Yang et al., 2016
*Mycobacterium tuberculosis*	Maitra et al., 2020
*Stenotrophomonas maltophilia*	Gomes et al., 2018

**TABLE 2:** Frequency of studied NSAIDs in thirty-two articles.

NSAID	Number of studies
Acetylsalicylic acid	Rosato et al., 2016; Yang et al., 2016; Belfield et al., 2017; Dotto et al., 2017; Fourie et al., 2017; Xu et al., 2017; Cai et al., 2018; Abidi et al., 2019; Leão et al., 2020; Lin et al., 2020; Santos et al., 2020; Wei et al., 2020
Diclofenac	Riordan et al., 2011; Yang et al., 2016; Abbas et al., 2020; Brilhante et al., 2020
Ibuprofen	Yang et al., 2016; Shah et al., 2018; Dai et al., 2019; Oliveira et al., 2019
Meloxicam	Soheili et al., 2015; She et al., 2018
Piroxicam	Soheili et al., 2015; Leão et al., 2020
Naproxen	Leão et al., 2020; Yan et al., 2020
Sodium salicylate	Belfield et al., 2017; Gerner et al., 2020
Celecoxib	Cai et al., 2018; Tzeng et al., 2020
Etodolac	Pereira et al., 2018
Carprofen	Maitra et al., 2020
Mefenamic Acid	Abidi et al., 2019

## Antibiofilm Activity of NSAIDs Against *Staphylococcus Aureus* and Selected Pathogenic Gram-Negative Species

*Staphylococcus aureus* has evolved intricate mechanisms to escape the immune system, efficiently invade and damage the host tissues, causing a multitude of infections (Kuehl et al., 2020). Its ability to form biofilms is a key mechanism that confers a survival advantage under harsh conditions. Attachment of *S*. *aureus* to medical implants and host tissues plays an important role in persistent chronic infections. Although biofilm production is a highly conserved mechanism in *S. aureus*, the composition and thickness of biofilms may differ among different strains (Kiedrowski et al., 2011; Vanhommerig et al., 2014). Methicillin-susceptible *S. aureus* (MSSA) and methicillin-resistant *S*. *aureus* (MRSA) may behave differently during infection, forming biofilms of varying biomass through different pathways (Lamret et al., 2020). Biofilm formation in MSSA is usually dependent on polysaccharide intercellular adhesin or polymeric *N*-acetyl-glucosamine, whereas MRSA strains frequently produce biofilms with a more proteinaceous matrix (Pozzi et al., 2012; Vanhommerig et al., 2014). Differences in biofilm phenotypes are also seen among the major MRSA clonal complexes that cause human infections, with the USA300 clone producing more robust and abundant biofilms under static and dynamic conditions than the other major clones (Vanhommerig et al., 2014).

Although there is evidence pointing to the activity of NSAIDs against *S. aureus* biofilms and other anti-virulence properties (e.g., inhibition of hemolysis and staphyloxanthin production; Abbas et al., 2020), most of these data were generated by *in vitro* studies using control laboratory bacterial strains, which may not necessarily indicate that these observations will be translated *in vivo*. These findings could also not be extrapolated to infections caused by different clonal complexes, given their significant differences in biofilm phenotypes (Vanhommerig et al., 2014). Thus, data from these *in vitro* experiments must be interpreted with caution whilst future studies investigate whether results are replicated in clinical isolates and *in vivo* models.

Studies testing the anti-biofilm potential of NSAIDs against other pathogenic bacteria have also been published in the last 5 years. *Pseudomonas aeruginosa* was the most studied Gram-negative pathogen (Table 1), with other species being scarcely tested. Gerner et al. conducted an interesting study involving *P*. *aeruginosa* clinical strains isolated from chronic wounds with different virulence profiles. Strains were evaluated using *in vitro* assays to produce biofilm, pyocyanin, siderophores, alkaline protease, elastase, and stapholytic protease. Exposure of these isolates to sodium salicylate, a sodium salt of salicylic acid, resulted in quorum-sensing inhibition and decreased virulence (Gerner et al., 2020). A previous report demonstrated that the growth of *P. aeruginosa* in the presence of salicylate reduced the production of extracellular polysaccharides required for biofilm formation (Pereira et al., 2018). Belfield et al. did not eradicate a mature biofilm applying ciprofloxacin or gentamicin 100 × MIC, with or without ASA or sodium salicylate or salicylic acid in high concentrations (200, 175, and 150 μg/ml, respectively). However, bacterial growth was inhibited by antibiotic–NSAID combinations (Belfield et al., 2017). Therefore, testing specific NSAIDs alone or associated with antibiotics and employing different concentrations on biofilms produced by high-risk and biofilm-producing bacterial clones could result in additional relevant data regarding biofilm control by NSAIDs (Cohen et al., 1993; Verma et al., 2018; Gerner et al., 2020).

### The Concentration of NSAIDs in Experimental Studies and Their Pharmacokinetics (PK) in Humans’ Trials

Because NSAIDs have been extensively tested in humans, pharmacokinetics data of these molecules can point toward whether or not concentrations tested *in vitro* against biofilms are achievable in the plasma, which would suggest a potential clinical application and drug repurposing. ASA concentrations ranging from 50 to 200 μg/ml, which are in the range frequently achieved by therapeutic ASA doses in humans, produces a significant level of anti-biofilm activity *in vitro* against *Candida albicans* (Alem and Douglas, 2004; Wei et al., 2020). Physiological concentrations of the drug was shown to reduce mature biofilms by 20–80% (*in vivo*), suggesting that ASA could have a significant inhibitory effect (Alem and Douglas, 2004). Salicylic acid at therapeutic concentrations was shown to reduce biofilms produced by *Candida* spp. (Alem and Douglas, 2004; Stepanović et al., 2004; Carvalho et al., 2010; Zhou et al., 2012), *Escherichia coli* (Kang et al., 1998; Chubiz and Rao, 2011; Vila and Soto, 2012), *P*. *aeruginosa* (Farber et al., 1995; Prithiviraj et al., 2005; El-Mowafy et al., 2014), and *Staphylococcus epidermidis* (Teichberg et al., 1993; Farber et al., 1995; Muller et al., 1998). Abbas et al. used a sub-MIC of diclofenac (1/4, 62.5 μg/ml) to investigate its inhibitory effect on different virulence factors of MRSA, including biofilm formation. Diclofenac exhibited a substantial reduction in biofilm formation compared to controls; the inhibition varied between 22.67 and 70% (Abbas et al., 2020). Diclofenac when tested against a *S. aureus* isolate demonstrated *in vitro* activity with a high MIC (2,000 μg/mL) and no detectable minimal bactericidal concentration (>2,000 μg/mL; Leão et al.). Even by applying a high diclofenac concentration (5 × MIC), anti-*S*. *aureus*-biofilm activity was not found. However, this high concentration of diclofenac had an effect in inactivating the *S*. *aureus* biofilm metabolism (inactivation percentages were between 80.4 and 86.7%). The authors observed a biofilm killing activity only when diclofenac was associated with kanamycin or tetracycline: at the established MIC (2,000 μg/ml) and 5 × MIC with kanamycin (low removal) and the MIC, 5 × MIC and 10 × MIC for tetracycline (low and moderate removal; Leão et al., 2020). Shah et al. observed a peak plasma concentration (Cmax) of 0.625 μg/ml for diclofenac, lower than the concentrations mentioned above. This pharmacokinetic study was performed with 30 volunteers who received 100 mg of diclofenac sodium (extended-release capsules; Shah et al., 2016). Of note, there is no safety data for doses above 150 mg once daily.

Dai et al. demonstrated positive results regarding the anti-biofilm formation and anti-quorum sensing activity of ibuprofen against *P. aeruginosa*. Furthermore, they found a concentration-dependent reduction in biofilm formation with rising drug concentrations (0, 50, 75, and 100 μg/mL). A previous PK study concerning intra-venous ibuprofen administration in children showed a mean Cmax = 35.8 μg/mL at a single dose of 8 mg/kg (Kauffman and Nelson, 1992).

Although the current PK data for ASA, diclofenac, and ibuprofen demonstrate that these molecules could achieve serum levels that are shown to have anti-biofilm activities *in vitro*, drug levels in tissues that are frequently infected by biofilms (bone and soft tissue in chronic osteomyelitis, valvar cardiac tissue in endocarditis, and urine or bladder tissue in recurrent urinary tract infections) need to be further evaluated.

## NSAIDs and Their Ability to Affect Tolerance and Antibiotic Resistance

Antibiotic tolerance and resistance are common mechanisms of survival within biofilm-embedded cells (Gerner et al., 2020). A proven unwanted side effect of salicylate, for example, is the modulation of antibiotic resistance (Cohen et al., 1993; Gustafson et al., 1999; Helal, 2012). In a biofilm, the high density of bacteria increases horizontal gene transfers, a common mechanism used by bacteria to disseminate antimicrobial resistance genes located in mobile genetic elements (Verma et al., 2018). Antibiotic tolerance is an ephemeral and reversible phenotypic state associated with differences in the physiology of cells according to their localization in the biofilm structure. Cells closer to the biotic or abiotic surfaces and thus more deeply located within the biofilm tend to be more deprived of oxygen and nutrients and join in a physiologically dormant state for which antimicrobials target key metabolic routes would not be active (Verma et al., 2018).

Experimental observations have demonstrated that exposure to NSAIDs can result in transcriptional adaptions that result in increased antibiotic resistance (Cohen et al., 1993; Gustafson et al., 1999; Helal, 2012; Verma et al., 2018). Verma et al. (2018) demonstrated that exposure of planktonic *E. coli* to sodium salicylate, ASA, acetaminophen, and ibuprofen could result in increased resistance to ciprofloxacin and tetracycline. Ibuprofen seems to be associated with low-level resistance phenotypes primarily associated with an increase in the efflux of antibiotics via the AcrAB-TolC efflux pump in a manner dependent on the transcriptional regulator MarA (Price et al., 2000; Helal, 2012). Increased antibiotic resistance following exposure to sodium salicylate or ASA, or acetaminophen is relatively higher when compared to ibuprofen and only partially dependent on MarA (Riordan et al., 2011). Other studies have shown that *E*. *coli* exhibits increased resistance to several antimicrobials (fluoroquinolones, ampicillin, cephalosporins, tetracycline, and chloramphenicol) in the presence of salicylate (Soheili et al., 2015; Belfield et al., 2017; Fourie et al., 2017; Pereira et al., 2018; She et al., 2018; Abidi et al., 2019; Dai et al., 2019). This drug also increases *S. aureus* resistance to fluoroquinolones and fusidic acid (Belfield et al., 2017; Cai et al., 2018; Oliveira et al., 2019; Abbas et al., 2020; Leão et al., 2020). Enhanced resistance to antimicrobials in the presence of salicylate has been attributed to the modification of membrane-associated proteins that results in reduced drug accumulation in the intracellular compartment and decreased susceptibility to a range of antimicrobials (Abidi et al., 2019). However, unlike ASA and ibuprofen, diclofenac did not induce antibiotic resistance in previous experimental research. Evidence regarding this successful outcome showed that diclofenac reduced the MIC for fluoroquinolones, oxacillin, and vancomycin against *S. aureus* (Price et al., 1999). One of the two clinical strains used in the experiment had a high ciprofloxacin MIC in the absence of diclofenac (MIC = 32), but a significant reduction in MIC in the presence of this drug at two different concentrations (MIC = 4; Price et al., 1999).

## Discussion

Recently, Leão et al. (2020) published an interesting study evaluating the *in vitro* activity of NSAIDs against *E. coli* and *S. aureus* biofilms. *In vitro* experiments showed that ASA diclofenac and ibuprofen have anti-biofilm activity in concentrations similar to those found in human pharmacokinetic studies (plasma concentrations). There are multiple suggested mechanisms of anti-bacterial and anti-biofilm actions of NSAIDs and they differ according to the species. For example, in *S. aureus*, NSAIDs seem to exert an antimicrobial or anti-biofilm activity by blocking AgrA-regulated virulence, inhibiting hemolysis, hindering staphyloxanthin production, and downregulating the expression *fnb*A and *ica*A genes, which are crucial in biofilm formation. ASA inhibits quorum-sensing in *P. aeruginosa* and salicylate reduces the production of extracellular polysaccharides required for biofilm formation (Hendrix et al., 2016; Pereira et al., 2018; Abbas et al., 2020).

Even though the available *in vitro* data can create some interest in the prospects of repurposing NSAIDs as anti-biofilm agents, more evidence from *in vivo* studies is required to determine whether the use of NSAIDs would translate into clinical efficacy in treating biofilm infections. Moreover, data regarding *in vitro* activity of NSAIDs against planktonic bacteria and biofilms are not consistent. According to the performed systematic search, only two studies have assessed NSAID activity against relevant pathogenic coagulase-negative staphylococci in the last 5 years (Pereira et al., 2018; Abidi et al., 2019). These organisms are the most common causes of healthcare-associated biofilm-related infections, like catheter-related bloodstream infections and prosthetic joint infections (De Vecchi et al., 2018; Hebeisen et al., 2019). Species like *S. epidermidis*, *Staphylococcus haemolyticus*, *Staphylococcus hominis*, are also important biofilm-forming microorganisms. *Enterococcus* spp. and some Gram-negative species have also been poorly studied and constitute a relevant pathogen in community and healthcare environments. *Enterococcus* spp. have been recognized as an important pathogen in chronic wound infections, mainly in chronic diabetes foot infections (Jneid et al., 2018; Carro et al., 2020). In the future, the NSAIDs could be an alternative tool to treat a delayed healing wound, clinical evidence of an active and mature biofilm. *Staphylococcus* spp. and *Enterococcus* spp. are also present in PJI, which could be linked to biofilm formation. Intracellular internalization of staphylococci, particularly “Small colony variant” strains, besides invasion and colonization of the lacuna-canalicular system of cortical bone are other causes of PJIs (Parvizi et al., 2013; Zoller et al., 2020). A clinical study explored if ASA could promote infection control for patients with periprosthetic joint infections (PJIs) (Wei et al., 2020). Regular ASA exposure was characterized as a prescription of ASA for >6 months prior to diagnosis of PJI and uninterrupted use during the PJI therapy at a dose ≧ 100 mg/day. Eighty-eight patients who met the PJI criteria were identified and incorporated in this study. Of these patients, 12 were taking ASA regularly during the infectious events. The Cox proportional hazard model analysis of time-to-infection resolution showed that the ASA was beneficial for PJIs ending (95% CI 1.018–4.757; *p* = 0.045; Wei et al., 2020). However, we do not know how many PJIs were biofilm-related and whether the anti-inflammatory effect of ASA proportionate a better outcome. Although it may represent an anti-biofilm effect of ASA, data regarding ASA and antimicrobial tissue concentrations, for example, are lacking. So, it is not possible to state that ASA works in this clinical setting. The *in vitro* experiments showing anti-biofilm inhibition or control with equal plasma serum concentrations identified in human pharmacokinetic studies should be reproduced, as well as new research employing other NSAIDs and distinct microorganisms (pointing out the clones of interest). Importantly, because of the positive data outcomes from PK *in vitro* studies, trials regarding NSAIDs levels on the tissues of biofilm-associated infections–like bone and soft tissue in chronic osteomyelitis, and valvar cardiac tissue in endocarditis–should be performed.

According to the outcomes of the study of Leão et al. (2020), the association between an antimicrobial and an NSAID–piroxicam or diclofenac or ASA employment with kanamycin or tetracycline–could be an interesting research field since this combination can result in biofilm removal. Unlike the study of Leão et al., Belfield et al. (2017) do not reach biofilm removal employing ciprofloxacin and gentamycin against *P. aeruginosa* and *S. aureus* strains isolated from clinical biofilm infections. Thus, the association mechanism of action between those drugs and the determination of the clonal lineages for each microorganism could clarify these discordant results.

Besides NSAIDs, alternative strategies and novel antibiofilm agents have been studied earlier, like nanoparticles, photodynamic therapy, natural compounds, anti-quorum sensing signaling molecules, matrix-degrading enzyme, and gene editing technique (CRISPR-CAS) (Sharma et al., 2019). Most of these tools, however, are also restricted to *in vitro* studies. So far, there is no place for NSAIDs in managing biofilm-related infections, even in non-life-threatening ones, like mild chronic wounds, despite the availability of topical commercial formulations of NSAIDs in high concentrations. Biofilms may be polymicrobial, which provides several advantages, such as passive resistance, metabolic cooperation, quorum sensing systems, an enlarged gene pool with more efficient DNA sharing, and many other synergies giving them a competitive advantage. Therefore, given the knowledge regarding biofilms in humans, data concerning NSAID concentrations, performance in distinct clones of pathogenic agents, and bacterial resistance is required to obtain sufficient evidence.

## Data Availability Statement

The raw data supporting the conclusions of this article will be made available by the authors, without undue reservation.

## Author Contributions

Both authors listed have made a substantial, direct and intellectual contribution to the work, and approved it for publication.

## Conflict of Interest

The authors declare that the research was conducted in the absence of any commercial or financial relationships that could be construed as a potential conflict of interest.

## Publisher’s Note

All claims expressed in this article are solely those of the authors and do not necessarily represent those of their affiliated organizations, or those of the publisher, the editors and the reviewers. Any product that may be evaluated in this article, or claim that may be made by its manufacturer, is not guaranteed or endorsed by the publisher.
