# Supplementary material for: Antimicrobial Activity of Non-steroidal Anti-inflammatory Drugs on Biofilm: Current Evidence and Potential for Drug Repurposing
Source: Front Microbiol. 2021 Jul 27;12:707629. doi: 10.3389/fmicb.2021.707629 (PMC8353384; doi:10.3389/fmicb.2021.707629)
Supplement: Supplementary file 1 [file Data_Sheet_1.docx]

**Appendix A**

Figure S1 – the Selection Strategy flow chart.

Table S1- Search strategy – PubMed

Table S2 – Search strategy – Scopus

**Figure S1**. Selection Strategy flow chart.

Records identified through database searching: 91 (PubMed)

Records identified through database searching: 187 (Scopus)

Records excluded: 193.

Duplicates: 44.

Records screened: 237.

44 full-texts articles assessed.

**Full-texts articles excluded: 12.**

- NSAID associated with drugs or materials, except antimicrobial agents.
- Non-human related pathogenic microorganisms.

**Studies included: 32.**

**Table S1**. Search strategy - PubMed.

| **Search strategy** | **Result** |
| --- | --- |
| ((("biofilm"[Title/Abstract]) OR ("biofilms"[Title/Abstract])) AND ((((((((((((((((((acetylsalicylic acid[Title/Abstract]) OR ("salicylate"[Title/Abstract])) OR ("celecoxib"[Title/Abstract])) OR ("diclofenac"[Title/Abstract])) OR ("ibuprofen"[Title/Abstract])) OR ("indomethacin"[Title/Abstract])) OR ("ketoprofen"[Title/Abstract])) OR ("naproxen"[Title/Abstract])) OR ("piroxicam"[Title/Abstract]))) OR ("etodolac"[Title/Abstract])) OR ("ketorolac"[Title/Abstract])) OR ("meloxicam"[Title/Abstract])) OR ("tenoxicam"[Title/Abstract])) OR ("non-steroidal anti inflammatories"[Title/Abstract])) OR ("non-steroidal anti-inflammatory"[Title/Abstract])) OR ("nsaid"[Title/Abstract])) OR (nsaids[Title/Abstract])). Filters: in the last 5 years | 91 |
| Searched on 10/30/2020 | |

**Table S2**. Search strategy – Scopus.

| **Search strategy** | **Result** |
| --- | --- |
| TITLE-ABS-KEY ( biofilm ) OR TITLE-ABS-KEY ( biofilms ) AND TITLE-ABS-KEY ( acetylsalicylic AND acid ) OR TITLE-ABS-KEY ( salicylate ) OR TITLE-ABS-KEY ( celecoxib ) OR TITLE-ABS-KEY ( diclofenac ) OR TITLE-ABS-KEY ( ibuprofen ) OR TITLE-ABS-KEY ( indomethacin ) OR TITLE-ABS-KEY ( ketoprofen ) OR TITLE-ABS-KEY ( naproxen ) OR TITLE-ABS-KEY ( piroxicam ) OR TITLE-ABS-KEY ( etodolac ) OR TITLE-ABS-KEY ( ketorolac ) OR TITLE-ABS-KEY ( meloxicam ) OR TITLE-ABS-KEY ( tenoxicam ) OR TITLE-ABS-KEY ( non-steroidal AND anti-inflammatories ) OR TITLE-ABS-KEY ( non-steroidal AND anti-inflammatory ) OR TITLE-ABS-KEY ( nsaid ) OR TITLE-ABS-KEY ( nsaids ) AND ( LIMIT-TO ( PUBSTAGE , "final" ) ) AND ( LIMIT-TO ( PUBYEAR , 2020 ) OR LIMIT-TO ( PUBYEAR , 2019 ) OR LIMIT-TO ( PUBYEAR , 2018 ) OR LIMIT-TO ( PUBYEAR , 2017 ) OR LIMIT-TO ( PUBYEAR , 2016 ) OR LIMIT-TO ( PUBYEAR , 2015 ) ) AND ( LIMIT-TO ( DOCTYPE , "ar" ) ) | *187 |
| Searched on 05/27/2021 | |

*We include the articles between 10/30/2015 and 10/30/2020.
